# Effect of Additional Terminal Residues on the Folding and Unfolding Dynamics of Cold Shock Protein

**DOI:** 10.1002/advs.202501369

**Published:** 2025-10-30

**Authors:** Dan Hu, Yang Wang, Huanjie Jiang, Hai Pan, Yunqiang Bian, Weitong Ren, Hu Chen, Zilong Guo, Yanwei Wang

**Affiliations:** ^1^ Department of Physics Wenzhou University Wenzhou 325035 China; ^2^ Center of Biomedical Physics Wenzhou Institute University of Chinese Academy of Sciences Wenzhou 325000 China; ^3^ Research Institute for Biomimetics and Soft Matter Fujian Provincial Key Lab for Soft Functional Materials Research Department of Physics Xiamen University Xiamen 361005 China

**Keywords:** additional terminal residues, cold shock protein (CSP), magnetic tweezers (MT), protein stability

## Abstract

Enhancing protein stability through modifications to the N‐ and C‐termini of natural proteins offers the distinct advantages of safety and cost‐effectiveness when compared to the denovo design of proteins. To explore the effect of additional residues at the termini on protein stability, single‐molecule magnetic tweezers are employed to examine the folding and unfolding dynamics of Cold Shock Protein (CSP) with various appended residues (LE‐CSP‐GS, KL‐CSP‐GS, KL‐CSP‐LE). The unfolding rate constant of the LE‐CSP‐GS is an order of magnitude faster than the others, while its folding rate constant decreased by more than an order of magnitude, resulting in upto ≈5 *k_B_T* bigger in folding free energy. Molecular dynamics (MD) simulations revealed that the stability differences are due to additional hydrogen bonds formed by residues K6 and E56. The combination of single‐molecule experiments and MD simulations indicates that additional residues at the termini can significantly affect protein stability and dynamics.

## Graphical abstract

Enhancing protein stability through N‐ and C‐terminal modifications presents a safe and cost‐effective strategy. We investigated this by combining single‐molecule magnetic tweezers experiments and molecular dynamics simulations to study the folding and unfolding dynamics of CSP with various appended residues (LE‐CSP‐GS, KL‐CSP‐GS, KL‐CSP‐LE). The LE‐CSP‐GS variant showed significantly faster unfolding and slower folding, leading to a substantial decrease in stability (∼5 kBT). Simulations revealed that stability differences originated from additional hydrogen bonds formed by residues K6 and E56. This work demonstrates that terminal modifications are a powerful tool for tuning protein stability and dynamics.

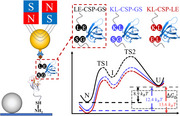

## 1 Introduction

From an evolutionary view, proteins originate as short, disordered peptide chains and evolve into molecules with both functional complexity and structural stability.^[^ ^1^ ^]^ The stability or function of a protein may be modulated by the addition of residues or modifications at the N‐ and C‐termini of its native structure.^[^ ^2^ ^]^ A comparison of 113 eukaryotic cytochrome c protein sequences reveals that the N‐ and C‐termini exhibit the greatest sequence variability, leaving the 3D structures to remain conserved across the superfamily.^[^ ^3^ ^]^ The cytochrome c also works as a model protein to study protein folding and unfolding dynamics.^[^ ^4^, ^5^ ^]^ However, the complex dynamics of the protein make it challenging to determine the impact of residues at the N‐ and C‐termini on protein stability.

The Ig domains on Titin, which govern the passive elasticity of the sarcomere and facilitate the conversion of chemical and mechanical signals,^[^ ^6^ ^]^ serve as protein folding model systems.^[^ ^7^ ^]^ Several residues without structure are present at the N‐ and C‐termini of Ig domains within the I‐band region of Titin.^[^ ^8^ ^]^ Understanding how these linkers influence the stability of the Ig domains is pivotal to their associated functions. However, the unpredictable nature of disulfide bonds and the interdependent stability of these Ig domains present significant challenges for single‐molecule force spectroscopy (SMFS) experiments aimed at assessing the effects of additional residues.^[^ ^9^, ^10^ ^]^

Another evolutionarily conserved protein is Cold Shock Protein (CSP), which is part of the cell's stress response system.^[^ ^11^ ^]^ The from *Thermotoga maritima* CSP, consisting of 66 amino acids, is a well‐studied model for protein folding and unfolding.^[^ ^12^, ^13^, ^14^, ^15^ ^]^ Given that different protein constructs and denaturation methods have been used in bulk and single‐molecule experiments, these well‐designed experiments give different folding free energy of CSP.

The folding and unfolding rates of CSP in denaturant solutions below and above ≈4 M were measured, respectively, resulting in a folding free energy to be Δ*G* = 10.4 *k_B_T*.^[^ ^16^ ^]^ In Differential Scanning Calorimetry (DSC) experiments, changes in enthalpy and heat capacity as a function of temperature were monitored to observe ensemble conformation changes of CSP, resulting in a folding free energy of 7.9 *k_B_T*.^17^ ^]^ SMFS denatures proteins by applying forces at the termini and monitoring the corresponding extension changes. The folding free energy of CSP is estimated to be ≈12.6 *k_B_T* by the equilibrium measurement at around critical force (≈6.6 pN).^[^ ^18^ ^]^

A difference of up to ≈5 *k_B_T* in folding free energy was observed between DSC and MT experiments. This discrepancy may arise from different models of folding and unfolding dynamics. For instance, linear fitting of the unfolding rate of CSP in 4–6 M guanidine hydrochloride was used to estimate the unfolding rate at zero denaturant, based on the assumption of a single barrier between the folded and unfolded states of CSP.

Alternatively, different restriction sites were used to insert the CSP gene into the plasmid, resulting in varying residues at the N‐ and C‐termini of CSP. In both denaturant and thermal denaturation experiments, CSP was expressed as described by Welker et al., with additional amino acids IH at the N‐terminus.^[^ ^12^ ^]^ To insert CSP between I27 domains in SMFS, additional residues KL and LE were added to the N‐ and C‐termini, respectively.^[^ ^18^ ^]^

Amino acid mutations in proteins, combined with experimental and molecular dynamics (MD) simulation approaches, are powerful tools to elucidate residue‐specific contributions to protein stability and dynamics. For instance, MD simulations of CSP revealed that hydrophobic core mutations (L40A and V26A) destabilize the protein by reducing core packing density and enhancing loop flexibility—a finding corroborated by atomic force microscopy (AFM) experiments.^[^ ^19^ ^]^ Comparative high‐temperature (500–600 K) MD studies between CSP from the thermophile *Bacillus caldolyticus* (Bc‐Csp) and from the mesophile *Bacillus subtilis* (Bs‐CspB) further demonstrated that slower unfolding kinetics in Bc‐Csp arise from stabilizing electrostatic networks involving Arg‐3/Glu‐46/Glu‐21/C‐terminal ion pairs. However, the impact of additional residues on CSP stability and dynamics remains underexplored.^[^ ^20^ ^]^

Using MT, we measured the force‐dependent folding and unfolding rates of four CSP construct: one with XhoI and BamHI restriction sites at the 5′ and 3′ ends of the DNA (LE‐CSP‐GS), one with HindIII and BamHI sites (KL‐CSP‐GS), one with HindIII and XhoI sites (KL‐CSP‐LE), and the native CSP without extra amino acid residues at both ends. The force‐dependent folding and unfolding rates of the engineered protein (LE‐CSP‐GS) and the native CSP were quantitatively similar. The unfolding rate constant of LE‐CSP‐GS was an order of magnitude faster than the others, while its folding rate constant decreased by more than an order of magnitude. MD simulations revealed that the stability divergence caused by the additional residues was due to the formation of extra hydrogen bonds by residues K and E.

## 2 Experimental Section and Simulation Results

### 2.1 Constant Loading Rate Measurement

**Figure 1:**
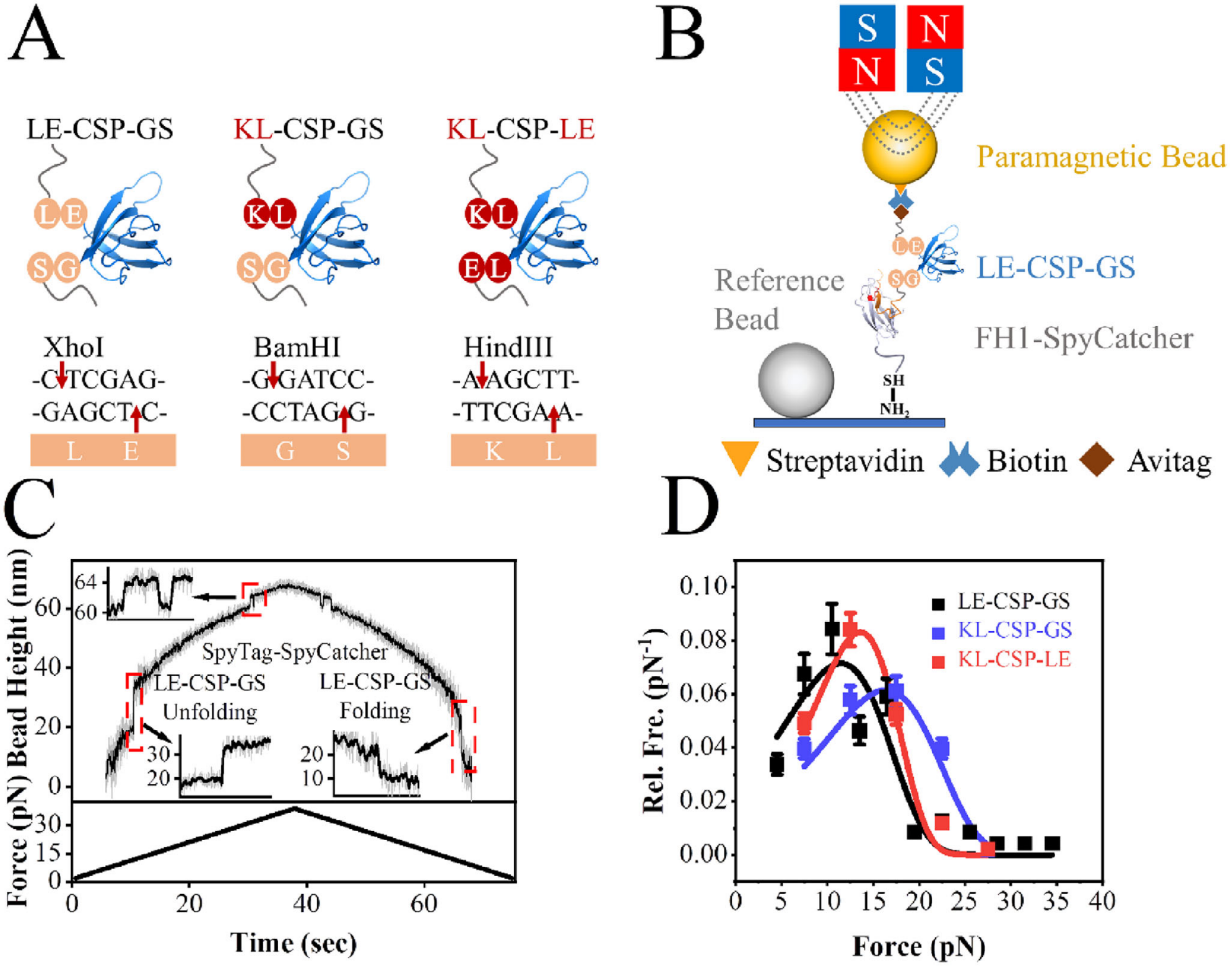
A) The restriction sites and corresponding residues. The gene sequence of CSP was insert into plasmid by XhoI, BamHI or HindIII, leaving the amino acids LE, GS, or KL at N‐ and C‐termini of CSP, respectively. B) Sketch of magnetic tweezers with a protein construct linked between a SpyCatcher covered glass surface and a Streptavidin covered paramagnetic bead. C) Force ramp measurement on CSP. The height of a tethered bead was recorded as the force increased with a constant loading rate of 1 pN s^−1^ from ≈2 to ≈42 pN, after that the force decreased with loading rate −1 pN s^−1^ from ≈42 to ≈2 pN. Raw data were recorded at a sampling rate of 200 Hz (gray) and smoothed using 20 points window (black). Inserts show the zoomed‐in figures of CSP unfolding and folding processes and equilibrium (un)zipping processes of SpyTag/SpyCatcher, respectively. D) The unfolding force distributions of LE‐CSP‐GS (black squares), KL‐CSP‐GS (blue squares) and KL‐CSP‐LE (red squares) at constant loading rate of 1 pN s^−1^. The distribution is fitted by Bell's model (Equation S2, Supporting Information) with $k_{u}^{0}=\left(2.4\pm 0.6\right)\times 10^{-2}s^{-1}$ and *x_u_* = 0.7 ± 0.1*nm* for LE‐CSP‐GS, $k_{u}^{0}=\left(1.2\pm 0.3\right)\times 10^{-2}s^{-1}$ and *x_u_* = 0.6 ± 0.1*nm* for KL‐CSP‐GS and $k_{u}^{0}=\left(1.2\pm 0.3\right)\times 10^{-2}s^{-1}$ and *x_u_* = 0.9 ± 0.1*nm* for KL‐CSP‐LE.

**Figure** **1**A illustrates the protein structures used in our single‐molecule experiments, and the corresponding restriction sites utilized for integrating the CSP gene into the plasmid. Apart from the residues introduced by the restriction sites, the amino acid sequences remain identical.

It tethered the CSP protein via a C‐terminal SpyTag anchored to a glass surface coated with SpyCatcher and an N‐terminal biotin conjugated to a streptavidin‐coated paramagnetic bead (M270) (Figure 1B).^[^ ^21^ ^]^ As illustrated in Figure 1C, the LE‐CSP‐GS tethers were subjected to stretching at a loading rate of 1 pN s^−1^, increasing the force from ≈2 to 42 pN, followed by relaxation from 42 to 2 pN at a rate of −1 pN s^−1^. An unfolding event occurred at ≈11.8 pN, marked by a single‐step extension of ≈15.5 nm, while a refolding event was detected at ≈4.2 pN, characterized by stepwise folding of ≈11.0 nm. At forces near 30 pN, unzipping‐zipping transitions of the SpyTag‐SpyCatcher complex were also observed, verifying that the bead was tethered by a single protein.^[^ ^22^ ^]^

Stretching cycles were conducted at a loading rate of 1 pN s^−1^, yielding at least 79 unfolding events at every construct. By fitting the unfolding force distributions using Equation S2 (Supporting Information),^[^ ^23^ ^]^ the most probable unfolding forces were determined to be ≈11.5 pN for LE‐CSP‐GS, 16.4 pN for KL‐CSP‐GS, and 13.6 pN for KL‐CSP‐LE. These force‐ramp measurements indicate that KL‐CSP‐GS exhibits a higher unfolding force compared to the other constructs. To further elucidate the influence of additional residues on the folding and unfolding kinetics, the force‐dependent rates of folding and unfolding for these CSP constructs should be measured.

### 2.2 Constant Force Equilibrium Measurement

**Figure 2:**
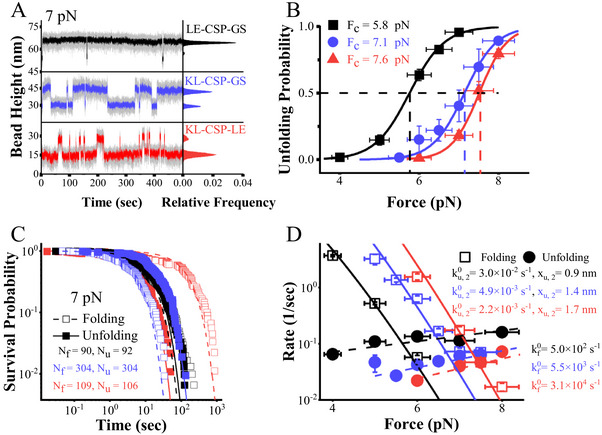
The equilibrium measurements of CSP at around critical forces. A) The bead height time course of LE‐CSP‐GS, KL‐CSP‐GS and KL‐CSP‐LE for 500 s at 7 pN. The black, blue, and red lines represent the smoothed results of LE‐CSP‐GS, KL‐CSP‐GS, and KL‐CSP‐LE, respectively, using a 0.1 s time window. The right panel shows corresponding relative frequency of the smoothed results and fitting with Gaussian function. B) Force‐dependent unfolding probabilities of these constructs. The average unfolding probabilities were determined from at least three independent tethers. C) The survival probability of native states and unfolded states at 7 pN. The solid and dash lines are exponential fitting results to determine *k_u_* and *k_f_*. D) Average folding rates (empty squares) and unfolding rates (solid circles) at forces of 4–8 pN are obtained from more than three independent tethers. The folding rates can be described using Arrhenius's law (Equation S7, Supporting Information). The unfolding rates are fitted with Bell's model to obtain the zero‐force unfolding rates and unfolding distances at low forces. Error bar of rates show the standard error of the mean, and force is estimated to have 5% uncertainty.

Using the constant‐force capabilities of magnetic tweezers, it directly measured equilibrium transitions between the Native state (N) and Unfolded state (U) states of CSP under constant forces. **Figure** **2**A illustrates a representative equilibrium measurement of the three CSP constructs at 7 pN. The right panel depicts Gaussian fits to the relative frequency histogram of the smoothed bead height, revealing two distinct peaks corresponding to the N and U states.

The force‐dependent unfolding probability *P_u_* (*f*) of CSPs at different constant forces was obtained from the Gaussian fitting of the histogram (Figure 2B), which can be fitted with Equation S3 (Supporting Information). The critical force of LE‐CSP‐GS was indeed smaller compared to KL‐CSP‐GS and KL‐CSP‐LE. The variations in folding probability underscore notable differences in the folding free energy of these constructs (LE‐CSP‐GS: ≈8.9 *k_B_T*; KL‐CSP‐GS: ≈12.4 *k_B_T*; KL‐CSP‐LE: ≈13.6 *k_B_T*). Folding and unfolding transitions were consistently observed across constant forces ranging from 4 to 8 pN.

With the Hidden Markov Model, the dwell time of equilibrium measurements obtained at different forces were got.^[^ ^24^ ^]^ To determine the unfolding and folding rates of CSP, the dwell time distributions for unfolding and folding events to calculate the survival probability as a function of time were analyzed, which was fitted to the exponential function *P*(*t*) = 1 − exp (− *kt*). Figure 2C shows the exponential fit for the three CSP constructs at 7 pN. The exponential fitting provides the unfolding and folding rates at each force level.(Figure S3, Supporting Information)

As depicted in Figure 2D, the force‐dependent unfolding rates of LE‐CSP‐GS (ranging from 4 to 8 pN), KL‐CSP‐GS (ranging from 5 to 8 pN), and KL‐CSP‐LE (ranging from 6 to 8 pN) were well‐described by Bell's model. For LE‐CSP‐GS, the fitting parameters (denoted with the subscript “2”) are as follows: $k_{u,2}^{0}=\left(3\pm 0.7\right)\times 10^{-2}s^{-1}$ and *x*_*u*,2_ = 0.9 ± 0.2*nm*. For KL‐CSP‐GS, the parameters are: $k_{u,2}^{0}=\left(4.9\pm 1.3\right)\times 10^{-3}s^{-1}$ and *x*_*u*,2_ = 1.4 ± 0.2*nm*, which closely resemble those of KL‐CSP‐LE: $k_{u,2}^{0}=\left(2.2\pm 0.7\right)\times 10^{-3}s^{-1}$ and *x*_*u*,2_ = 1.7 ± 0.2*nm*. The unfolding rate of LE‐CSP‐GS was 6 and 13 times higher than that of KL‐CSP‐GS and KL‐CSP‐LE, respectively, while the difference between KL‐CSP‐GS and KL‐CSP‐LE was indistinctive.

The folding rates of these three constructs exhibit considerable sensitivity to the presence of additional residues. The force‐dependent folding rate of LE‐CSP‐GS was at least an order of magnitude lower than those of the other two constructs. Moreover, a fivefold difference in folding rates was observed between KL‐CSP‐GS and KL‐CSP‐LE. Considering that CSP demonstrates distinct sensitivities at both high and low forces,^[^ ^18^ ^]^ further investigation into the impact of additional residues on CSP at high forces was warranted.

### 2.3 Force‐Dependent Unfolding Rates at Big Forces

**Figure 3:**
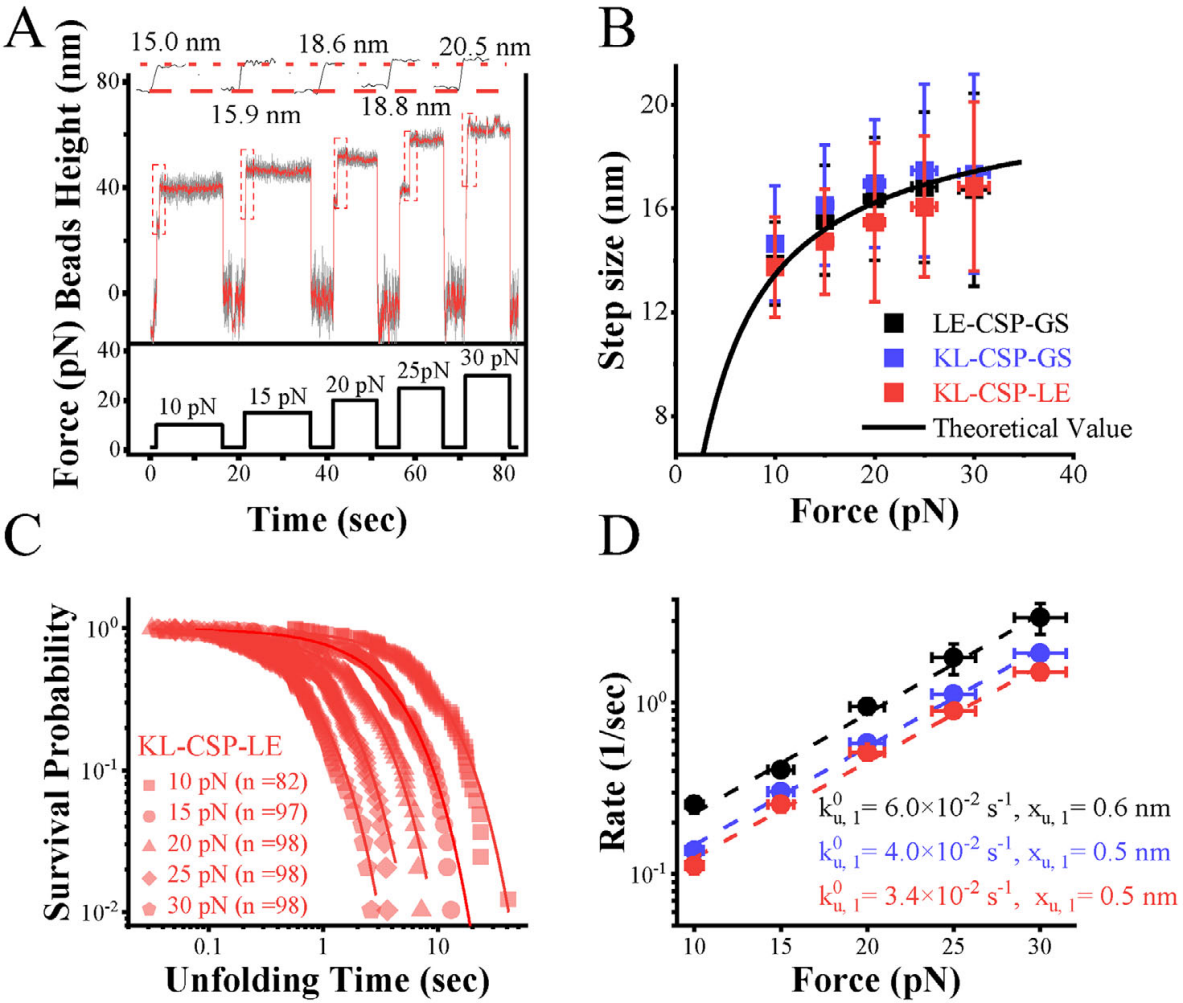
The force‐jump measurement of CSP constructs. A) Force‐jump measurement of the unfolding courses of KL‐CSP‐LE at 10 to 30 pN. B) The unfolding stepsize of three CSP constructs. The force‐dependent unfolding stepsize of CSP equals extension of the unfolded peptide minus extension of native state, Δ*x*(*f*) = *x_chain_* (*f*) − *x_CSP_* (*f*). C) The survival probability of native states and unfolded states of KL‐CSP‐LE from 10 to 30 pN. D) Unfolding rates from force jump experiment for three CSP constructs from 10 to 30 pN. The unfolding rates of LE‐CSP‐GS at 10–30 pN (black line), KL‐CSP‐GS at 10–30 pN (blue line), and KL‐CSP‐LE at 10–30 pN (red line) are fitted with Bell's model to determine $k_{u,1}^{0}$ and *x*_*u*,1_.

It performed force‐jump experiments to measure the unfolding rates of the three CSP constructs at forces ranging from 10 pN to 30 pN. As illustrated in **Figure** **3**A, the force was initially held at ≈1 pN to maintain CSP in N. Subsequently, the force was increased from ≈1 pN to higher levels, spanning between 10 and 30 pN, during which unfolding events were recorded. To determine the force‐dependent step sizes and unfolding rates, at least 82 unfolding events were obtained at each force.

The force‐dependent step sizes in bead height, observed during transitions between the N and U across various force‐jump experiments, were extracted and analyzed (Figure 3B; Figures S5‐S7, Supporting Information). The resulting force‐step size curves can be accurately described by modeling U as a Worm‐Like Chain model and N as a Freely Jointed Chain model.^[^ ^25^, ^26^ ^]^

The unfolding rate, *k_u_* (*f*), in force‐jump measurements was determined by exponential fitting to the survival probability of CSP's native state (Figure 3C; Figure S3, Supporting Information). Bell's model was employed to fit the high‐force unfolding rates, and fitting parameters are shown in Figure 3D. The unfolding rates follow the trend LE‐CSP‐GS>KL‐CSP‐GS>KL‐CSP‐LE under identical force conditions. These findings suggest that the additional residues exert a similar influence on TS1 during CSP unfolding.

### 2.4 Free‐Energy Landscapes of LE‐CSP‐GS, KL‐CSP‐GS and KL‐CSP‐LE

**Figure 4:**
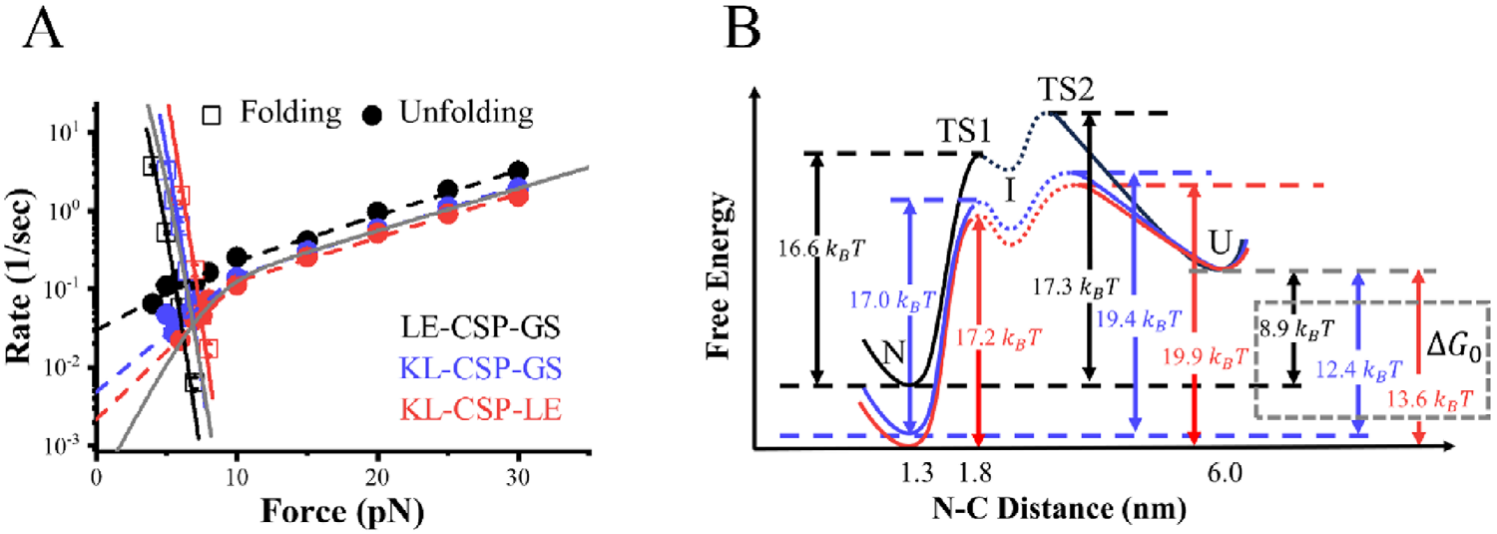
A) The force‐dependent folding rates (empty squares) and unfolding rates (solid circles) of three CSP from 4 to 30 pN. The slopes of the two fitting lines of unfolding rates give distinct unfolding distances *x*_*u*,1_ = 0.6 *nm* and *x*_*u*,2_ = 0.9 *nm* for LE‐CSP‐GS, *x*_*u*,1_ = 0.5 *nm* and *x*_*u*,2_ = 1.4 *nm* for KL‐CSP‐GS, *x*_*u*,1_ = 0.5 *nm* and *x*_*u*,2_ = 1.7 *nm* for KL‐CSP‐LE. The gray line in the figure represents data obtained from ref. [18]. B) Free energy landscape of CSP at zero force. With free energy of U as a reference, the energy of N is obtained by Equation S9 (Supporting Information). Energy of TS1 and TS2 is calculated from fitting parameters of Bell's model parameters, $k_{u,1}^{0}\;$ and $k_{u,2}^{0}$.

**Figure** **4**A illustrates the unfolding and folding rates of CSP across a force range of 4–30 pN. In our previous studies, a three‐state (N‐I‐U) model was utilized to characterize the force‐dependent unfolding kinetics of CSP across a broad force spectrum (4–30 pN).^[^ ^27^ ^]^ Although the CSP exhibits distinct force‐sensitivity regimes (4–8 pN and 10–30 pN), prompting our hypothesis of an intermediate state (I) bridging two transition barriers. However, the transient intermediate state cannot be directly observed by unfolding steps or force‐extension curves. The three‐state model's introduction of two additional fitting parameters (compared to the two‐state model) further exacerbates concerns about overfitting (Figure S13, Tables S1, and S2, Supporting Information). Therefore, the force‐dependent unfolding rates for the three constructs were fitted with Bell's model, employing distinct parameters ($k_{u,1}^{0}$, *x*_*u*,1_, $k_{u,2}^{0}$, and *x*_*u*,2_) for forces above and below 10 pN, respectively. Given the very similar force‐dependent folding and unfolding rates observed for LE‐CSP‐GS and the native CSP (Figure S11, Supporting Information), the experimental results for the three engineered protein constructs are compared in Figure 4.

Assuming the intrinsic unfolding rate of CSP was k* ≈ 10^6^ *s* ^−1^,^[^ ^28^ ^]^ the energy barriers (ΔG*) can be calculated using the equation $k_{u}^{0}=k^{*}\exp\left(-\Delta G^{*}/k_{B}T\right)$. As depicted in Figure 4, using the unfolded state as the reference, the free energy landscapes of LE‐CSP‐GS, KL‐CSP‐GS, and KL‐CSP‐LE were plotted as functions of the N‐C distance. The N‐C distance of CSP, determined to be 1.3 nm, represents the distance between the α‐carbon atoms of the residues at the N‐terminus and C‐terminus in its native structure (PDB: 1G6P). The N‐C distance of TS1 was derived by combining *x*_*u*,1_ with the N‐C distance of N, yielding 1.8 nm. Similarly, the N‐C distances of TS2 were estimated as 2.2 nm, 2.7 nm, and 3.0 nm for LE‐CSP‐GS, KL‐CSP‐GS, and KL‐CSP‐LE, respectively.

From the force‐dependent folding and unfolding rates, the energy difference between N and U state, Δ*G*_0_, was calculated using Equation S8 (Supporting Information) to be 8.9 *k_B_T* for LE‐CSP‐GS, 12.4 *k_B_T* for KL‐CSP‐GS, and 13.6 *k_B_T* for KL‐CSP‐LE. These findings suggest that the additional KL residues at the N‐terminus elevate the energy of TS2 and increase the folding free energy of CSP.

### 2.5 Molecular Dynamics Simulation

To further determine the interactions between the additional residues and original structure of CSP, it performed Molecular Dynamics simulations. First, the structure of CSP with additional residues at N‐ and C‐termini were predicted by AlphaFold3‐server,^[^ ^29^ ^]^ and no additional interaction was observed.

To further investigate the interaction between the additional residues and CSP structures, MD simulations were performed using GROMACS. The predicted CSP structures were immersed in a water box to simulate the protein buffer environment. System preparation utilized the CHARMM36m force field alongside GROMACS 2023.^[^ ^30^, ^31^ ^]^ After energy minimization and stepwise equilibration under NVT and NPT conditions, a 1000 ns unbiased all‐atom molecular dynamics simulation was performed in the NPT ensemble. Long‐range electrostatics were calculated using the PME method, and van der Waals interactions were handled up to 1.2 nm.

Equilibrium simulations of three CSP constructs across three independent 1000 ns MD simulations revealed a potential interaction between the additional lysine (K6) of KL at the N‐terminus and the residue glutamate (E56).

**Figure 5:**
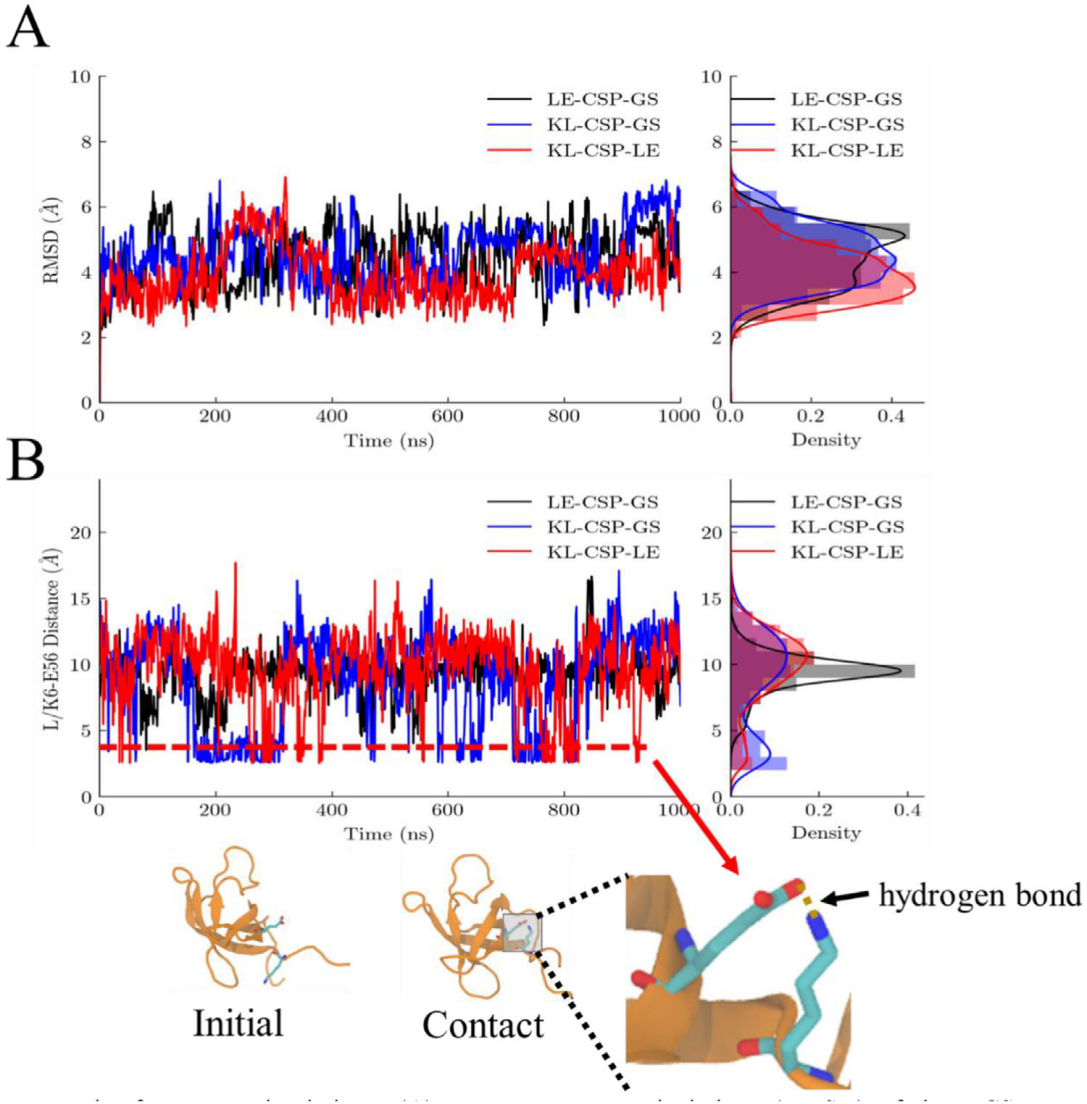
Results from MD simulations. A) Root mean‐square deviations (RMSD) of three CSP construct independent 1000 ns MD simulations (LE‐CSP‐GS, black line; KL‐CSP‐GS, blue line; KL‐CSP‐LE, red line). The right panel illustrates the density distribution. B) Atomic distance between the Leu6‐Cζ or Lys6‐Nζ and Glu56‐Oε atoms: LE‐CSP‐GS (black line), KL‐CSP‐GS (blue line), and KL‐CSP‐LE (red line).The right panel illustrates the density distribution. Insert shows the KL‐CSP‐LE initial structure with Lys6 and Glu56 separated by >10 Å, where yellow dashed lines and arrows indicate transient contacts and potential hydrogen bond formation between these residues.

The root mean‐square deviations of the three CSP constructs are shown in **Figure** **5**A. All three replicate simulations showed consistent small deviations (2–6Å) for each construct (Figure 5A; Figure S13, Supporting Information). Figure 5B shows the statistical distances between the additional K6 or L6 and E56 of CSP. As the inset shows the KL‐CSP‐LE initial structure with Lys6 and Glu56 separated by >10 Å, where yellow dashed lines and arrows indicate transient contacts and potential hydrogen bond formation between these residues. The specific atomic distance between the Lys6‐Nζ and Glu56‐Oε atoms remained within the hydrogen‐bonding threshold (< 3.5 Å) for 14%. In the remaining two repeated simulations, it was 8% and 7% (Figure S13C,D, Supporting Information). In contrast, this interaction was not observed in the LE‐CSP‐GS construct. Our analysis confirms that the formation of the K6‐E56 salt bridge‐strengthened hydrogen bond was a highly robust and reproducible feature. This interaction was believed to enhance the unfolding barrier and increase the folding free energy of CSP.

## 3 Summary and Discussion

Previous studies on CSP have extensively characterized effect of hydrophobic core interactions, loop flexibility, and electrostatics on (un)folding and stability by mutating amino acid sequences or comparing homologous sequences.^[^ ^19^, ^31^ ^]^ In this work, we reveals a critical yet overlooked factor: the influence of additional terminal residues, introduced by digestion site, on protein stability and dynamics. Previous well‐designed experiments, often yield inconsistent folding free energy (7.9–12.6 *k_B_T*) and dynamic profiles. AFM experiments revealed a significantly higher number of intermediate states during CSP unfolding compared to MT measurements.^[^ ^18^, ^32^ ^]^ By streamline the protein construct, we demonstrate that N‐ or C‐terminal residues significantly affect CSP (un)folding rate and folding free energy.

To investigate the effect of additional residues on the stability of CSP, we measured its folding and unfolding rates across constructs with various residues appended to the N‐ and C‐termini: LE‐CSP‐GS, KL‐CSP‐GS, and KL‐CSP‐LE. Given that CSP's unfolding rates display differing sensitivities under high and low force conditions, we analyzed the unfolding rates of these constructs at both small forces (4–8 pN) and big forces (10–30 pN). At small forces, the unfolding rate of LE‐CSP‐GS is ≈6 and 13 times higher than those of KL‐CSP‐GS and KL‐CSP‐LE, respectively. At higher forces, the unfolding rates follow the trend: LE‐CSP‐GS>KL‐CSP‐GS>KL‐CSP‐LE.

Distinct unfolding barriers, TS1 and TS2, govern the force‐dependent unfolding rates of CSP at big and small forces, respectively. TS1 is particularly sensitive to the presence of KL or LE residues at the N‐terminus but remains unaffected by GS or LE residues at the C‐terminus. Conversely, the energy of TS2, which dominates folding and unfolding rates at forces below 10 pN, is influenced by residues at both the N‐ and C‐termini.

The folding free energy of these constructs was determined through equilibrium measurements under constant forces. The folding probabilities of CSP at constant forces yielded folding free energies of 8.9 *k_B_T*, 12.4 *k_B_T*, and 13.6 *k_B_T* for LE‐CSP‐GS, KL‐CSP‐GS, and KL‐CSP‐LE, respectively.

Notably, the folding free energy of KL‐CSP‐LE is ≈5 *k_B_T* higher than that of LE‐CSP‐GS. Additionally, the difference in folding free energy of CSP measured using DSC and MT is also ≈5 *k_B_T*. These findings demonstrate that protein stability can be significantly enhanced by the addition of residues to the N‐ or C‐termini.

Molecular dynamics simulations suggest a potential interaction between the additional K6 and E56 residues, which may play a role in stabilizing TS1 and thus reducing the unfolding rates at high forces. However, sampling the structures of TS2 is still a challenge in MD simulations, which are more relaxed and exhibit higher free energy relative to the N and TS1.

Protein stability is often enhanced through ligand binding, with binding affinity largely influenced by the ligand's concentration. In these single‐molecule experiments, the additional residues at the N‐ and C‐termini of CSP exhibit an effective concentration estimated to be in the millimolar range.^[^ ^33^ ^]^ Enhancing protein stability by appending residues to the N‐ or C‐termini provides notable advantages in safety and cost‐efficiency compared to de novo protein design or amino acid sequence mutations.

## Conflict of Interest

The authors declare no conflict of interest.

## Supporting information



Supporting Information

## Data Availability

The data that support the findings of this study are available from the corresponding author upon reasonable request.
