# Supplementary material for: Effect of Additional Terminal Residues on the Folding and Unfolding Dynamics of Cold Shock Protein
Source: Adv Sci (Weinh). 2025 Oct 30;12(48):e01369. doi: 10.1002/advs.202501369 (PMC12752555; doi:10.1002/advs.202501369)
Supplement: Supplementary file 1 — Supporting Information [file ADVS-12-e01369-s001.docx]

Supporting Information

Effect of Additional Terminal Residues on the Folding and Unfolding Dynamics of Cold Shock Protein

Dan Hu, Yang Wang, Huanjie Jiang, Hai Pan, Yunqiang Bian, Weitong Ren, Hu Chen*, Zilong Guo*, Yanwei Wang*

Dan Hu, Yanwei Wang, Huanjie Jiang

Department of Physics, Wenzhou University, Wenzhou 325035, China

Dan Hu, Yang Wang, Hu Chen, Hai Pan, Yunqiang Bian, Weitong Ren, Zilong Guo

Center of Biomedical Physics, Wenzhou Institute, University of Chinese Academy of Sciences, Wenzhou 325000, China

Hu Chen

Research Institute for Biomimetics and Soft Matter, Fujian Provincial Key Lab for Soft Functional Materials Research,Department of Physics, Xiamen University, Xiamen 361005, China

E-mail: chenhu@xmu.edu.cn, guozilong@ucas.ac.cn, wangyw@wzu.edu.cn.

Keywords: Additional Terminal Residues, Cold Shock Protein (CSP), Magnetic Tweezers (MT), Protein Stability

(Dan Hu and Yang Wang contributed equally to this work.)

1. Protein sequences

In the following, the first three sequences correspond to the CSPs, with the N- and C-termini of CSP highlighted in bold. The differences among these three sequences are indicated in red to emphasize the variations. Based on the sequence of CSP, we predict the complex structure of CSP by the latest and highly accurate deep learning-based protein structure prediction tool AlphaFold3-server[1, 2]. Furthermore, native CSP construct without additional residues as a control to assess the impact of terminal additional residues on protein stability.

LE-CSP-GS sequences:

Avitag-GGGSG- **LE**-

MRGKVKWFDSKKGYGFITKDEGGDVFVHWSAIEMEGFKTLKEGQVVEFEIQEGKKGPQAAHVKVVE(CSP)-**GS**- GGGSG-Spytag

KL-CSP-GS sequences:

Avitag-GGGSG- **KL**-

MRGKVKWFDSKKGYGFITKDEGGDVFVHWSAIEMEGFKTLKEGQVVEFEIQEGKKGPQAAHVKVVE(CSP)-**GS**- GGGSG-Spytag

KL-CSP-LE sequences:

Avitag-GGGSG- **KL**-

MRGKVKWFDSKKGYGFITKDEGGDVFVHWSAIEMEGFKTLKEGQVVEFEIQEGKKGPQAAHVKVVE(CSP)-**LE**- GGGSG-Spytag

Native CSP sequences:

Avitag-GGGSG-

MRGKVKWFDSKKGYGFITKDEGGDVFVHWSAIEMEGFKTLKEGQVVEFEIQEGKKGPQAAHVKVVE(CSP)- GGGSG-Spytag

2. Experiment

A. Protein construct preparation

The recombinant protein constructs were created by inserting gene sequences of CSP cloned from E. coli using PCR technology into the vector pET151 through restriction sites. To produce biotinylated proteins, plasmids of pET151 and pBirA (biotin ligase expression plasmid) were transformed into the E. coli strain BL21 (DE3). The selected transformant on LB plates was cultured in LB medium supplemented with chloramphenicol, ampicillin, and D-biotin at 37 °C until the optical density of the bacterial cells reached 0.6~0.8. Protein expression was induced by adding 0.5 mM isopropyl β-D-thiogalactopyranoside and incubating for 12 h at 18 °C. The cells were harvested by centrifugation and lysed by sonication in a buffer containing 50 mM Tris, 500 mM NaCl, 10% glycerol, 5 mM imidazole, 5 mM 2-mercaptoethanol, and pH 8.0. The target proteins were purified using Ni-NTA Sefinose resin (Sangon Biotech) and Superdex 200 (GE Healthcare) following the manufacturer’s protocol. Finally, the protein was snap-frozen in liquid nitrogen and stored at -80 °C.

B. Sample preparation for magnetic tweezers.

Functionalized coverslips were used to make flow chambers and anchor the protein construct. Coverslips were first roughly cleaned in detergent (5% Decon90 in DI water) by sonication for 15 min, rinsed by DI water, and dried in oven at 70 °C. Then the coverslips were treated by oxygen plasma cleaner for 15 min, followed by incubation in methanol solution of 1% 3-aminopropyltriethoxysilane (APTES, cat. A3648, Sigma) for 1 h. These amino-modified coverslips were then washed by methanol and DI water, and dried in oven at 70°C for 40 min. Flow chambers were made by sandwiching a piece of functionalized coverslip and another piece of coverslip with parafilm in between. To eliminate spatial drift during experiment, Polybead Amino Microspheres (cat. 17145, Polysciences) with diameter of 3.0 µm were flowed into chamber and incubated for 20 min to get stuck on the coverslip. After rinsing by 200 µL 1xPBS buffer, 1% Sulfo-SMCC (SE 247420, Thermo Science) in DI water was flowed into the chamber and incubated for 20 min. After that, SpyCatcher protein in PBS was flowed into chamber to coat the functionalized surface for 1 h. 1% BSA 1x PBS buffer was flowed into chamber and incubated overnight at 4 °C to passivate the surface. Before single molecule experiment, chambers were incubated in PBS with around 1 nM protein for 20 min. Finally, Streptavidin-coated paramagnetic beads Dynabead M270 (cat. 65305, Invitrogen) were flowed into the chamber to form protein tethers.

The homemade magnetic tweezers were built around an inverted microscope that utilized double antiparallel magnetic rods to apply stretching forces on proteins for studying their force-dependent folding and unfolding dynamics. For detailed design information regarding the magnetic tweezers, please refer to our previous publication[3, 4].

3. Theoretical modeling

Bell’s model is employed to describe force-dependent unfolding rate $k_{\text{u}}\left( f \right)$ of the two-state proteins[5, 6]:

$$\begin{aligned} k_{\text{u}}\left( f \right)=k_{\text{u}}^{0}\exp\left( fx_{\text{u}}/k_{B}T \right)\#\left( S1 \right) \end{aligned}$$

Where, $k_{\text{u}}^{0}$ denotes the unfolding rate at zero force, $x_{\text{u}}$ the unfolding distance, $k_{B}$ the Boltzmann constant, and $T$ the absolute temperature.

Bell’s model is employed to simulate the unfolding force distribution obtained from constant pulling speed experiments, and the unfolding force distribution $P_{\text{f}}\left( f \right)$ at constant loading rate $r$ is[7]:

$$\begin{aligned} P_{\text{f}}\left( f \right)=\frac{k_{u}^{0}}{r}\exp\left[ \frac{fx_{\text{u}}}{k_{\text{B}}T}+\frac{k_{u}^{0}k_{\text{B}}T}{x_{\text{u}}r}\left( 1-exp\frac{fx_{\text{u}}}{k_{\text{B}}T} \right) \right]\#\left( S2 \right) \end{aligned}$$

[8, 9].

At a small force which is close to critical force, the probability of CSP staying at unfolded state, $P_{\text{u}}\left( f \right)$, was obtained from equilibrium measurements,

$$\begin{aligned} P_{\text{u}}\left( f \right)=\frac{1}{\exp\left[ -\left( f-f_{\text{c}} \right)\Delta x/\left( k_{\text{B}}T \right) \right]+1}\#\left( S3 \right) \end{aligned}$$

where $f_{\text{c}}$ denotes the critical force at which $P_{\text{u}}\left( f_{\text{c}} \right)=50\%$; $\Delta x$ the extension changes; $k_{\text{B}}$ Boltzmann constant, and $T$ the absolute temperature.

Folded CSP being modeled as a solid body, its extension along force direction $x_{\text{CSP}}$ changes due to orientation fluctuation:

$$\begin{aligned} x_{\text{CSP}}\left( f \right)=l_{\text{0}}\coth\left( \frac{fl_{\text{0}}}{k_{\text{B}}T} \right)-\frac{k_{\text{B}}T}{f}\#\left( S4 \right) \end{aligned}$$

Similar to the native state, we consider the transition state of protein as a solid body, and only its orientation fluctuation changes its extension $x_{\text{TS}}\left( f \right)$ in the direction of force, which can be described by:

$\begin{aligned} x_{\text{TS}}\left( f \right)=l_{\text{TS}}\coth\left( \frac{fl_{\text{TS}}}{k_{\text{B}}T} \right)-\frac{k_{\text{B}}T}{f}\#\left( S5 \right) \end{aligned}$

where, $l_{\text{TS}}$ represents the size of transition state, which equals the combination of N-C distance of N and the unfolding distance $x_{\text{u}}$.

As shown in **Figure S5**, **S6, S7 (F)**, The force-extension curves of unfolded CSP peptide can be well described by the worm-like chain (WLC) model:

$$\begin{aligned} \frac{fA}{k_{\text{B}}T}=\frac{x_{\text{chain}}}{L}+\frac{1}{4\left( 1-\frac{x_{\text{chain}}}{L} \right)^{2}}-\frac{1}{4}\#\left( S6 \right) \end{aligned}$$

where A denotes the persistence length of 0.8 nm, $x_{\text{chain}}$ the extension, and L the contour length of peptide.

The Arrhenius's law is employed to fit the force-dependent folding rates, $k_{\text{f}}^{0}$,

$$\begin{aligned} k_{\text{f}}\left( f \right)=k_{\text{f}}^{0}\exp\left( -\int_{0}^{f} x_{\text{f}}\left( f^{'} \right)df^{'}/k_{\text{B}}T \right)\#\left( S7 \right) \end{aligned}$$

where, $x_{\text{f}}\left( f \right)=x_{\text{chain}}\left( f \right)-x_{\text{TS}}\left( f \right)$ represents the extension difference between the TS and the unfolded state, while $k_{\text{f}}^{0}$ signifies the folding rate at zero force.

The force-dependent protein folding free energy $\Delta G\left( f \right)$ is given by:

$$\begin{aligned} \Delta G\left( f \right)=\Delta G_{0}-\int_{0}^{f} \Delta x\left( f^{'} \right) \text{d}f^{'}\#\left( S8 \right) \end{aligned}$$

where $\Delta G_{0}$ is folding free energy at zero force, the force-dependent stepsize is $\Delta x=x_{\text{chain }}-x_{\text{CSP}}$. Force-dependent protein folding free energy $\Delta G\left( f \right)$ of CSP can also be determined by the ratio between folding rate $k_{\text{f}}\left( f \right)$ and unfolding rate $k_{\text{u}}\left( f \right)$ at the same force $f$:

$$\begin{aligned} \Delta G\left( f \right)=k_{\text{B}}T\ln\frac{k_{\text{f}}\left( f \right)}{k_{\text{u}}\left( f \right)}\#\left( S9 \right) \end{aligned}$$

The unfolding and folding rates at zero force, $k_{u}^{0}$ and $k_{\text{f}}^{0}$, are obtained from extrapolation on force-dependent folding and unfolding rates, and $\Delta G_{0}$ can be determined directly.

As illustrated in Figure 4 (main text), the force-dependent unfolding rates of the three CSP constructs exhibit distinct sensitivities to low forces (<9 pN) and high forces (>10 pN). Following the approach of Christopher A. Pierse and Olga K. Dudko[6], we employed a three-state (N-I-U) model to fit the unfolding kinetics across a force range of 4–30 pN:

$$\begin{aligned} k_{\text{u}}\left( f \right)=\left[ 1-\left( 1+\frac{k^{\text{NI}}}{k^{\text{IN}}+k^{\text{IU}}} \right)^{-1} \right]k^{\text{IU}}\#\left( S10 \right) \end{aligned}$$

where $k^{\text{NI}}$ denotes transition rate from N to I, $k^{\text{IN}}$ transition rate from I to N, and $k^{\text{IU}}$ transition rate from I to U. For the transient hidden sate I, we assumed that $k^{\text{NI}}\ll k^{\text{IN}}$ and $k^{\text{NI}}\ll k^{\text{IU}}$. Then **Equation S10** can be simplified to:

$$\begin{aligned} k_{\text{u}}\left( f \right)=\frac{k^{\text{NI}}k^{\text{IU}}}{k^{\text{IN}}+k^{\text{IU}}}\#\left( S11 \right) \end{aligned}$$

This result is the same to rate equation of enzymatic chemical reaction[5]. As illustrated in **Figure S14** and **Table 1,** **Table 2**, the model incorporates two additional parameters compared to Bell's model when fitting force-dependent unfolding data at both low and high forces. This increase in parameters may lead to overfitting, thereby obscuring the mechanistic contributions of extra residues at the N- and C-termini.

4. Molecular dynamics simulation

The predicted structure of truncated CSP was solvated in a water box of $8\times8\times8 nm^{3}$, respectively. Configuration and topology files were generated using the CHARMM-GUI server[10-13]. All molecular dynamics simulations were performed exclusively with the CHARMM36m force field[14]. In addition to the structure of CSP, the simulation system included about 15,650 water molecules, 45 sodium, and 44 chloride ions (mimicking the 150 mM NaCl present in protein buffer), resulting in 48,221 atoms. A 1000 ns all-atom MD simulation trajectory was generated using GROMACS 2023[15] at 303 K using a time step 2 fs. The cubic periodic boundary condition was used during the simulations and the van der Waals interaction was switched off from 1 to 1.2 nm. The Particle Mesh Ewald (PME) method calculated the long-range electrostatic interactions. Energy minimization was done using the steepest descent algorithm, followed by a 0.4 ns NVT (constant particle number, volume, and temperature) and a 20 ns NPT (constant particle number, pressure, and temperature) equilibration simulation by gradually decreasing force restraints from 1000 $\text{kJ mo}\text{l}^{\text{-1}}\text{ n}\text{m}^{\text{-2}}$ to 400 $\text{mo}\text{l}^{\text{-1}}\text{ n}\text{m}^{\text{-2}}$ (for NVT stage) and 400 $\text{kJ mo}\text{l}^{\text{-1}}\text{ n}\text{m}^{\text{-2}}$ to 40 $\text{mo}\text{l}^{\text{-1}}\text{ n}\text{m}^{\text{-2}}$ (for NPT stage). All force restraints were removed after the equilibration steps, and the 1000 ns unbiased molecular dynamics simulation was performed in the NPT ensemble for each system.

5. Influence of pH and salt concentration

To test the effects of pH and salt concentration changes on proteins, we used the KL-CSP-LE construct as an example.

**Figure S12(A-C)** show the folding/unfolding rates of KL-CSP-LE in Tris buffer pH = 6, 7, 8. To regulate the ionic composition of the solution, a buffer consisting of 10 mM Tris and 150 mM NaCl was prepared, with the pH adjusted using HCl. Using magnetic tweezers to measure the equilibrium transition at force range of 6-7.5 pN. To minish the effect introduced by heterogeneity of magnetic beads, the equilibrium experimental data at different pH values were obtained from a same tether. As shown in **Figure S12 (A)**, approximately sixfold difference in folding rates of KL-CSP-LE is observed between PBS buffer and Tris buffer, and the folding rate of KL-CSP-LE remains unchanged across different pH conditions.

As shown in **Figure S12 (B)**, the unfolding rate of KL-CSP-LE in Tris buffer at pH 8 is approximately two- to threefold higher than at pH 6 or 7. From **Figure S12 (C)**, unfolding rates at forces ranging from 10 to 30 pN yielded the same result under the corresponding conditions.

To investigate the effect of salt concentration, the measurements were performed in 0, 100, and 1000 mM NaCl solutions. To minimize the introduction of additional ions, 10 mM Tris at pH 7.5 was used to benchmark the pH of the PBS solution, with the salt concentration gradually increased on this basis. In **Figure S12 (D)**, the folding rate of KL-CSP-LE in Tris buffer without NaCl was the same as in PBS buffer (red solid line) and an order of magnitude faster than in 1000 mM NaCl solutions. Compare **Figure S12 (E)** and **(F)**, at both small (5.5–7.5 pN) and big (10–30 pN) forces, showing no significant difference between low and high salt concentrations.

6. Figures


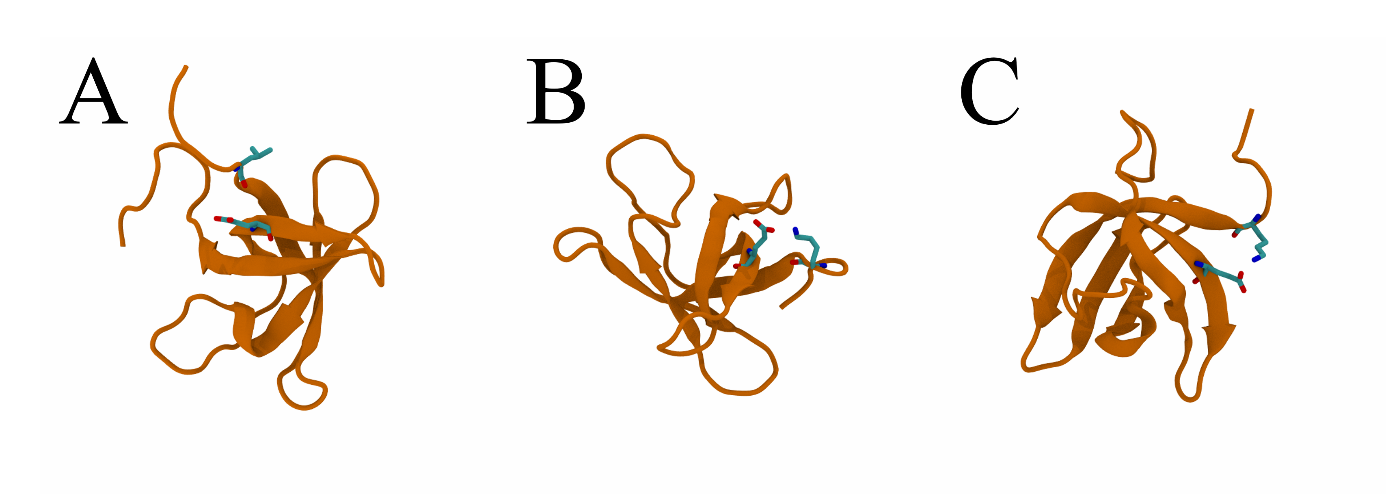


Figure S1 The structures of CSP with additional residues. The structures of CSP (A) LE-CSP-GS. (B) KL-CSP-GS. (C) KL-CSP-LE, were predicted by AlphaFold3. The residues at 6th and 56th in the construct are represented by stick. Other identical parts are all shown in brown.

Figure S2 Force-ramp measurements on (A) LE-CSP-GS, (B) KL-CSP-GS, and (C) KL-CSP-LE. The beads height were recorded (top panel) when force increased with a loading rate of 1 pN/s from $\sim$2 to $\sim$42 pN and decreased with loading rate -1 pN/s from $\sim$42 to $\sim$2 pN (bottom panel). Raw data were recorded at a sampling rate of 200 Hz (gray) and smoothed using 50 ms time window. Insets show the zoomed-in figures of CSPs unfolding and folding processes and equilibrium (un)zipping processes of SpyTag/SpyCatcher[3], respectively.

Figure S3 Equilibrium measurements of (A) LE-CSP-GS (B) KL-CSP-GS (C) KL-CSP-LE at constant forces. Raw data of beads height were recorded at 200 Hz (gray). The black, blue, and red lines represent the smoothed results of beads height, using a 0.1 s time window.

Figure S4 The survival probability of native states (solid) and unfolded states (empty) of (A, B) LE-CSP-GS, (C, D) KL-CSP-GS and (E, F) KL-CSP-LE. The solid and dash lines are exponential fitting results to determine $k_{u}$ and $k_{f}$, using the single exponential Equation $P=1-e^{-kt}$.

Figure S5 Force-step size curve of unfolded LE-CSP-GS from native state and unfolding stepsizes. (A-E) Histogram of transition step size at 10, 15, 20, 25, 30 pN are fitted by Gaussian functions (black line). (F) Step size of unfolded peptide is obtained by adding the extension of native state with the unfolding stepsize. Error bar of step size is given by the standard deviation of stepsizes, and force is estimated to have 5% uncertainty. Force-step size curve is fitted with a worm-like chain model (black line) with persistence length, A = 0.8 nm, and counter length, L = 23.8 nm.

Figure S6 Force-step size curve of unfolded KL-CSP-GS from native state and unfolding stepsizes. (A-E) Histogram of transition step size at 10, 15, 20, 25, 30 pN are fitted by Gaussian functions (blue line). (F) Step size of unfolded peptide is obtained by adding the extension of native state with the unfolding stepsize. Error bar of step size is given by the standard deviation of stepsizes, and force is estimated to have 5% uncertainty. Force-step size curve is fitted with a worm-like chain model (black line) with persistence length, A = 0.8 nm, and counter length, L = 23.8 nm.

Figure S7 Force-step size curve of unfolded KL-CSP-LE from native state and unfolding stepsizes. (A-E) Histogram of transition step size at 10, 15, 20, 25, 30 pN are fitted by Gaussian functions (red line). (F) Step size of unfolded peptide is obtained by adding the extension of native state with the unfolding stepsize. Error bar of step size is given by the standard deviation of stepsizes, and force is estimated to have 5% uncertainty. Force-step size curve is fitted with a worm-like chain model (black line) with persistence length, A = 0.8 nm, and counter length, L = 23.8 nm.

Figure S8 Force-dependent unfolding rates and folding rates of LE-CSP-GS. Different colors represent folding/unfolding rates measured from different tethers, while black show the mean value. (A) Unfolding rates measured by constant force equilibrium measurement from 4 to 9pN (Fig. S2). Black line shows the fitting result of Bell's model from 4 to 9 pN with $x_{u,2}=0.9 nm$ and $k_{u,2}^{0}=3\times{10}^{-2} s^{-1}$. (B) Unfolding rates from force jump measurements at force 10-30 pN, and fitting with Bell's model gives an unfolding distance, $x_{u,1}=0.6 nm$ and $k_{u,1}^{0}=6\times{10}^{-2} s^{-1}$. (C) Folding rates from constant force equilibrium measurements at 4-7 pN. **Equation S5** with the size of folding transition state$l_{TS}=2.2 nm$ fits the measured folding rates well.

Figure S9 Force-dependent unfolding rates and folding rates of KL-CSP-GS. Different colors represent folding/unfolding rates measured from different tethers, while blue show the mean value. (A) Unfolding rates measured by constant force equilibrium measurement from 5 to 8 pN. Blue line shows the fitting result of Bell's model from 5 to 8 pN with $x_{u,2}=1.4 nm$ and $k_{u,2}^{0}=4.9\times{10}^{-3} s^{-1}$. (B) Unfolding rates from force jump measurements at force 10-30 pN, and fitting with Bell's model gives an unfolding distance, $x_{u,1}=0.5 nm$ and $k_{u,1}^{0}=4.0\times{10}^{-2} s^{-1}$. (C) Folding rates from constant force equilibrium measurements at 5-8 pN. **Equation S5** with the size of folding transition state$l_{TS}=2.7 nm$ fits the measured folding rates well.

Figure S10 Force-dependent unfolding rates and folding rates of KL-CSP-LE. Different colors represent folding/unfolding rates measured from different tethers, while red show the mean value. (A) Unfolding rates measured by constant force equilibrium measurement from 6 to 8 pN. Red line shows the fitting result of Bell's model from 6 to 8 pN with $x_{u,2}=1.7 nm$ and $k_{u,2}^{0}=2.2\times{10}^{-3} s^{-1}$. (B) Unfolding rates from force jump measurements at force 10-30 pN, and fitting with Bell's model gives an unfolding distance, $x_{u,1}=0.5 nm$ and $k_{u,1}^{0}=3.4\times{10}^{-2} s^{-1}$. (C) Folding rates from constant force equilibrium measurements at 6-8 pN. **Equation S5** with the size of folding transition state $l_{TS}=3.0 nm$ fits the measured folding rates well.

Figure S11 Force-dependent folding rates (empty squares) and unfolding rates (solid circles) of LE-CSP-GS (black) and native CSP (orange) from 4 to 30 pN. Folding rates were fitted with Arrhenius’ law (**Equation S7**, solid lines). Unfolding rates were fitted with Bell's model (**Equation S1**, dashed lines) to derive the zero-force unfolding rates ($k_{u}^{0}$) and unfolding distances ($x_{u}$) at small (4–8 pN) and big (10–30 pN) force regimes respectively. Data obtained from more than three independent tethers. Error bars are the standard error of the mean of rates and 5% uncertainty of force. (A) Equilibrium measurements for LE-CSP-GS and native CSP constructs. (B) Unfolding rates from force jump experiments for LE-CSP-GS and native CSP constructs.

Figure S12 The folding rates (empty squares) and unfolding rates (solid circles) of KL-CSP-LE under (A-C) varying pH (pH = 6 (blue), 7(yellow), 8(pink)) and (D-F) sodium ion concentrations (0 mM(blue), 100 mM(yellow), 1000 mM(pink) ). The folding/unfolding rates for 6.0-7.5 pN and unfolding rate for 10-30 pN are measured on the same tether under each condition, respectively. Force-dependent folding(solid red line) and unfolding (dashed red line) of KL-CSP-LE in 1x PBS solution (red line) are taken from **Figure 2D** and **Figure 3D**. And the data on the same graph at different pH values and sodium ion concentrations were from a same tether.

**
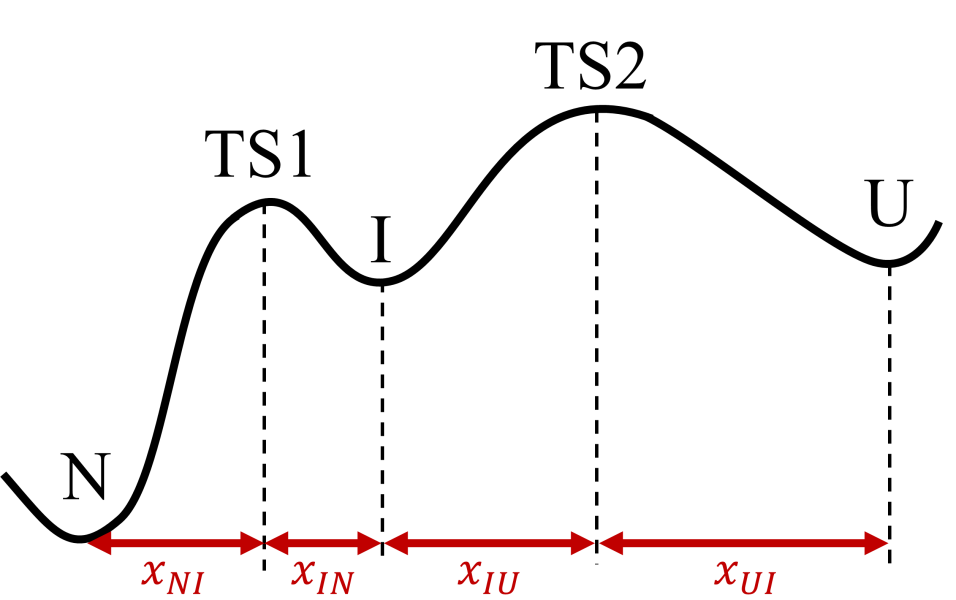
**

Figure S13 One-dimensional free energy landscape with two distinct transition states (TS1, TS2) and an intermediate state (I). The energy barriers are estimated from $k_{u}^{0}$in Bell's model and a pre-factor of ${10}^{6}s^{-1}$.

Figure S14 Force-dependent unfolding rate of three CSP constructs are fitted with three-state (N-I-U) model across a broad force spectrum (4–30 pN). The N-I-U model has clearly exhibited overfitting on the force-dependent unfolding rate and fitting parameters are not unique. Corresponding parameters are shown in **Table 1** and **Table 2**.

**
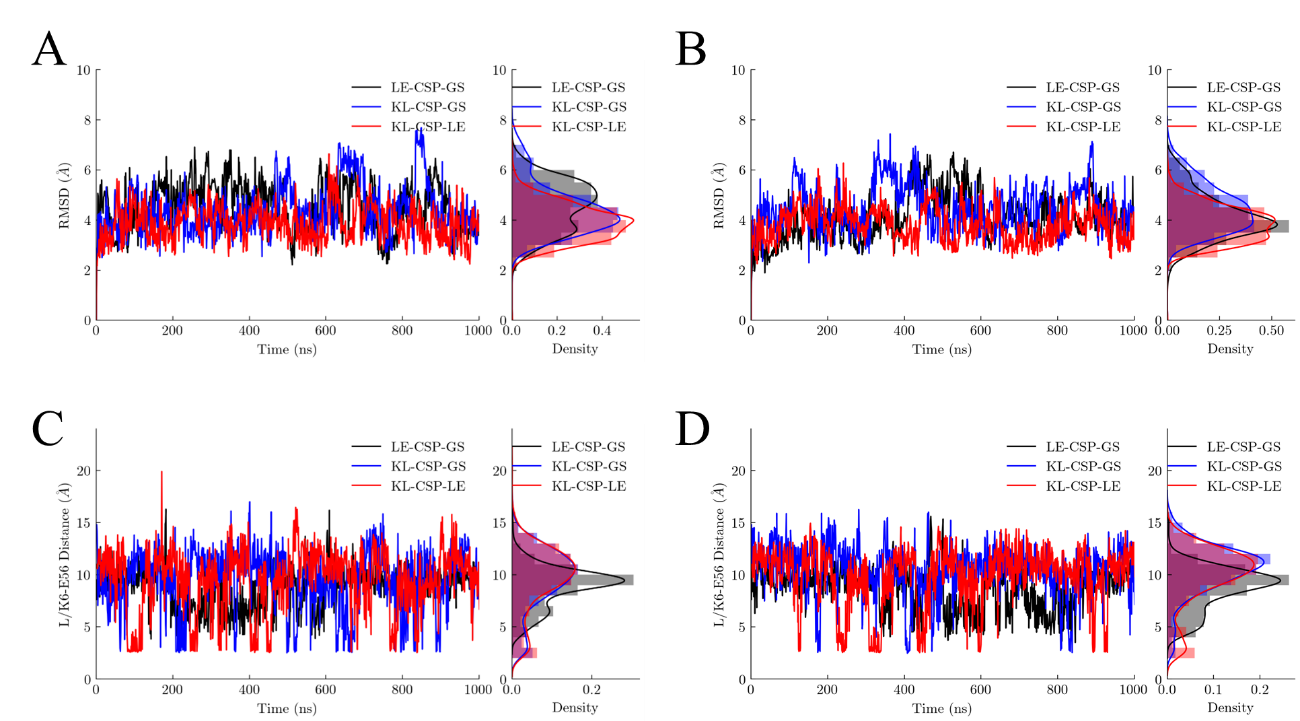
**

Figure S15 Results from multiple independent MD simulations. (A)(B) Root mean-square deviations (RMSD) of three CSP construct independent 1000 ns MD simulations (LE-CSP-GS, black line; KL-CSP-GS, blue line; KL-CSP-LE, red line). The right panel illustrates the density distribution. (C)(D) Atomic distance between the Leu6-Cζ or Lys6-Nζ and Glu56-Oεatoms: LE-CSP-GS (black line), KL-CSP-GS (blue line), and KL-CSP-LE (red line).The right panel illustrates the density distribution.

7.Tables

**Table S1.** Parameters from N-I-U model fitting: CSP (Hong et al., 2021) [16] vs. Our three CSP constructs.

|  | NIU model | | | | | |
| --- | --- | --- | --- | --- | --- | --- |
|  | $k_{NI}^{0}$ ($s^{-1}$) | $x_{NI}$ (nm) | $k_{IN}^{0}$ ($s^{-1}$) | $x_{IN}$ (nm) | $k_{IU}^{0}$ ($s^{-1}$) | $x_{IU}$ (nm) |
| 2xI_27_-CSP-2xI_27_[16] | $4.6\times{10}^{-2}$ | 0.51 | $3.8\times{10}^{4}$ | 1.30 | 230 | 1.3 |
| LE-CSP-GS | $4.7\times{10}^{-7}$ | 13.32 | $1.1\times{10}^{-3}$ | 15.99 | 130.35 | 3.23 |
| KL-CSP-GS | $5.7\times{10}^{-2}$ | 0.48 | 2.10 | -1.41 | 0.13 | $5.3\times{10}^{-2}$ |
| KL-CSP-LE | $4.6\times{10}^{-2}$ | 0.48 | 6.08 | -0.57 | $5.2\times{10}^{-2}$ | 1.90 |

**Table S2.** Parameters from Bell's model fitting: CSP (Hong et al., 2021) [16] vs. Our three CSP constructs.

|  | Bell’s model | | | |
| --- | --- | --- | --- | --- |
|  | $k_{u,1}^{0}$ ($s^{-1}$) | $x_{u,1}$ (nm) | $k_{u,2}^{0}$ ($s^{-1}$) | $x_{u,2}$ (nm) |
| 2xI_27_-CSP-2xI_27_[16] | $3.2\times{10}^{-2}$ | 0.55 | $2\times{10}^{-4}$ | 3.1 |
| LE-CSP-GS | $6.0\times{10}^{-2}$ | 0.6 | $3\times{10}^{-2}$ | 0.9 |
| KL-CSP-GS | $4.0\times{10}^{-2}$ | 0.5 | $4.9\times{10}^{-3}$ | 1.4 |
| KL-CSP-LE | $3.4\times{10}^{-2}$ | 0.5 | $2.2\times{10}^{-3}$ | 1.7 |

**References**

1. Valds-Tresanco, M.S., et al., *Gmx_Mmpbsa: A New Tool to Perform End-State Free Energy Calculations with Gromacs.* Journal of Chemical Theory and Computation, 2021. **17**(10): p. 6281––6291.

2. Jumper, J., et al., *Highly Accurate Protein Structure Prediction with Alphafold.* Nature, 2021. **596**(7873): p. 583––589.

3. Guo, Z., et al., *Spytag/Spycatcher Tether as a Fingerprint and Force Marker in Single-Molecule Force Spectroscopy Experiments.* Nanoscale, 2021. **13**(25): p. 11262––11269.

4. Chen, H., et al., *Improved High-Force Magnetic Tweezers for Stretching and Refolding of Proteins and Short DNA.* Biophysical Journal, 2011. **100**(2): p. 517––523.

5. Bell, G.I., *Models for the Specific Adhesion of Cells to Cells: A Theoretical Framework for Adhesion Mediated by Reversible Bonds between Cell Surface Molecules.* Science, 1978. **200**(4342): p. 618––627.

6. Pierse, C.A. and O.K. Dudko, *Distinguishing Signatures of Multipathway Conformational Transitions.* Physical Review Letters, 2017. **118**(8): p. 088101.

7. Schlierf, M., H. Li, and J.M. Fernandez, *The Unfolding Kinetics of Ubiquitin Captured with Single-Molecule Force-Clamp Techniques.* Proceedings of the National Academy of Sciences, 2004. **101**(19): p. 7299––7304.

8. Torsten, S., et al., *Dynamic Force Spectroscopy of Single DNA Molecules.* Proceedings of the National Academy of Sciences, 1999. **96**(20): p. 11277–11282.

9. Sulchek, T.A., et al., *Dynamic Force Spectroscopy of Parallel Individual Mucin1--Antibody Bonds.* Proceedings of the National Academy of Sciences, 2005. **102**(46): p. 16638––16643.

10. Abramson, J., et al., *Accurate Structure Prediction of Biomolecular Interactions with Alphafold 3.* Nature, 2024: p. 1––3.

11. Jo, S., et al., *Charmm-Gui Membrane Builder for Mixed Bilayers and Its Application to Yeast Membranes.* Biophysical Journal, 2009. **97**(1): p. 50––58.

12. Jo, S., et al., *Charmm-Gui: A Web-Based Graphical User Interface for Charmm.* Journal of Computational Chemistry, 2008. **29**(11): p. 1859––1865.

13. Wu, E.L., et al., *Charmm-Gui Membrane Builder toward Realistic Biological Membrane Simul Ations*. Wiley Online Library.

14. Lee, J., et al., *Charmm-Gui Input Generator for Namd, Gromacs, Amber, Openmm, and Charmm/Openmm Simulations Using the Charmm36 Additive Force Field.* Biophysical Journal, 2016. **110**(3): p. 641a.

15. Huang, J., et al., *Charmm36m: An Improved Force Field for Folded and Intrinsically Disordered Proteins.* Nature Methods, 2017. **14**(1): p. 71––73.

16. Hong, H., et al., *Two Energy Barriers and a Transient Intermediate State Determine the Unfolding and Folding Dynamics of Cold Shock Protein.* Communications Chemistry, 2021. **4**(1): p. 156.
